# Ras-Induced miR-146a and 193a Target Jmjd6 to Regulate Melanoma Progression

**DOI:** 10.3389/fgene.2018.00675

**Published:** 2018-12-18

**Authors:** Viviana Anelli, Anita Ordas, Susanne Kneitz, Leonel Munoz Sagredo, Victor Gourain, Manfred Schartl, Annemarie H. Meijer, Marina Mione

**Affiliations:** ^1^Cibio, University of Trento, Trento, Italy; ^2^Institute of Biology, Leiden University, Leiden, Netherlands; ^3^Physiological Chemistry, Biocenter, University of Würzburg, Würzburg, Germany; ^4^Institute of Toxicology and Genetics, Karlsruhe Institute of Technology, Karlsruhe, Germany; ^5^Faculty of Medicine, University of Valparaiso, Valparaíso, Chile; ^6^Comprehensive Cancer Center, University Clinic Würzburg, Würzburg, Germany; ^7^Hagler Institute for Advanced Study and Department of Biology, Texas A&M University, College Station, TX, United States

**Keywords:** zebrafish, cancer models, microRNA, Jmjd6, ras, melanoma, miR-146a, miR-193a

## Abstract

Ras genes are among the most commonly mutated genes in human cancer; yet our understanding of their oncogenic activity at the molecular mechanistic level is incomplete. To identify downstream events that mediate ras-induced cellular transformation *in vivo*, we analyzed global microRNA expression in three different models of Ras-induction and tumor formation in zebrafish. Six microRNAs were found increased in Ras-induced melanoma, glioma and in an inducible model of ubiquitous Ras expression. The upregulation of the microRNAs depended on the activation of the ERK and AKT pathways and to a lesser extent, on mTOR signaling. Two Ras-induced microRNAs (miR-146a and 193a) target Jmjd6, inducing downregulation of its mRNA and protein levels at the onset of Ras expression during melanoma development. However, at later stages of melanoma progression, *jmjd6* levels were found elevated. The dynamic of Jmjd6 levels during progression of melanoma in the zebrafish model suggests that upregulation of the microRNAs targeting Jmjd6 may be part of an anti-cancer response. Indeed, triple transgenic fish engineered to express a microRNA-resistant Jmjd6 from the onset of melanoma have increased tumor burden, higher infiltration of leukocytes and shorter melanoma-free survival. Increased *JMJD6* expression is found in several human cancers, including melanoma, suggesting that the up-regulation of Jmjd6 is a critical event in tumor progression.

The following link has been created to allow review of record GSE37015: http://www.ncbi.nlm.nih.gov/geo/query/acc.cgi?token=jjcrbiuicyyqgpc&acc=GSE37015.

## Introduction

Activating mutations in the *RAS* genes or in other members of the ras-signaling pathways are very common in cancer^1^ and recent deep sequencing data of cancer genomes^2^ suggest that these mutations are important primers of malignancies. Still, the initial molecular events following activation of the pathways downstream of Ras are extremely difficult to study *in vivo.* Transgenic models, where the expression of the oncogene leads to cancer development in a reproducible manner provide a suitable experimental system for addressing the complexity of cellular transformation in live animals. The oncogenic versions of the human RAS genes (KRAS, HRAS, and NRAS) have been the first and most successful drivers of cancer in transgenic mice (Chin et al., 1999a,b; Johnson et al., 2001; Malumbres and Barbacid, 2003). This ability of ras oncogenes to initiate and maintain cancer has been related to global molecular and epigenetic changes at early stages of transformation. Among the targets of oncogenes, microRNAs are well-suited to sustain global changes of cellular functions. Changes in several protein levels may be regulated by a single or just a few microRNAs and a number of microRNAs have been found deregulated in cancer (Harrandah et al., 2018). Yet, very few studies have investigated their roles at the onset of transformation as possible “global effectors” of oncogenesis. In this study we have investigated the link between Ras-induced transformation and microRNA expression, using genetically tractable zebrafish models where the expression of a constitutively active *HRAS^G12V^* allele leads to the development of different cancer types. We found that activated Ras signaling promotes the rapid increase of six microRNAS. Interestingly, two of these microRNAs target the same gene, Jmjd6, a jumonjiC domain protein with at least two reported functions: histone arginine demethylation (Chang et al., 2007) and mRNA splicing regulation (Webby et al., 2009). Results reported here indicate that Jmjd6 is a critical player in zebrafish melanoma development and that at least two Ras-induced microRNAs antagonize Jmjd6 activation.

## Results

### Dynamic Regulation of MicroRNAs by RAS Activation

**FIGURE 1:**
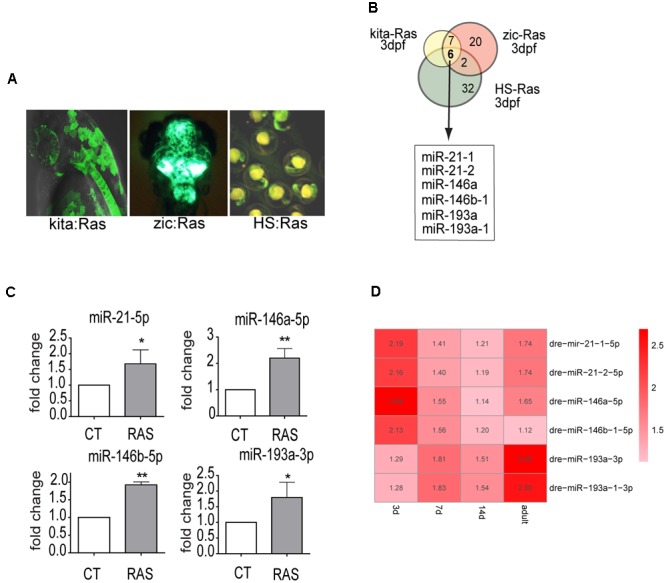
miR-21, miR-146a/b, and miR-193a are upregulated by oncogenic RAS in transgenic cancer models. **(A)** Zebrafish models used in the study of Ras-dependent microRNAs. Green fluorescence denotes the expression of the eGFP-fused oncogene. In Kita:Ras GFP labels transformed melanocytes and notochord; in zic:Ras GFP marks ras-expressing brain cells; in HS-Ras, eGFP-Ras is expressed in whole embryos. For full description of transgenic lines, see text. **(B)** Diagram depicting the overlap between the three sets of upregulated microRNAs. **(C)** Taqman QPCR analysis of miR-21, miR-146a/b, and miR-193a expression levels in 3 dpf kita-RAS larvae compared to control (CT) larvae. The error bar represents the SEM of a triplicate experiment. **(D)** Heatmap representation of microarray analysis of miR-21, miR-146a/b, and miR-193a expression at different stages of melanoma progression. Distinct precursor sequences and genomic loci that express identical mature sequences are named on the form miR-21-1 and miR-21-2. Lettered suffixes denote closely related mature sequences. –5p and –3p indicate the 5′ and 3′ arm respectively. ^∗^*P* ≤ 0.05 and ^∗∗^*P* ≤ 0.01.

To identify miRNAs that are regulated by oncogenic Ras from the earliest stages of transformation, we used a custom Agilent microarray (see M&M). We profiled miRNA expression in transgenic zebrafish overexpressing *HRAS^G12V^* in melanocytes (Santoriello et al., 2010), in brain cells (Mayrhofer et al., 2017) and ubiquitously (Santoriello et al., 2009) (**Figure 1A**). Six miRNAs (miR-21.1, miR- 21.2, miR-146a, miR-146b-1, miR-193a, miR-193a-1) were up-regulated (log2FC > 1.2 and *p*-value < 0.01, see **Supplementary Table S1**) in all three transgenic models at 3 day post-fertilization (dpf) and in 7 dpf melanoma, whereas no commonly down-regulated miRNAs were found (**Figure 1B** and **Supplementary Table S1**, highlighted rows).

This study, we focused on the melanoma model, and to clarify the potential roles of the upregulated microRNAs in melanoma progression, we analyzed microRNA expression levels at four time points (3, 7, and 14 dpf and in adult tumors, **Supplementary Table S1**) during melanoma progression. Next, we designed Taqman probes for the active strand (-5p) of miR21, miR-146a and miR-146b and for the active strand (-3p) of miR-193a and validated the expression levels of the six upregulated miRNAs by qPCR in the melanoma model (**Figure 1C**). The expression of the six microRNA genes showed dynamic patterns: one was upregulated up to 7 dpf (miR-146b-1-5p), while the others were upregulated to different extents up to adult melanoma (**Figure 1D** and **Supplementary Table S1**). To further validate the dependence of these microRNAs on ras we used drugs that target different pathways downstream of ras. Next, expression levels of miR-21-5p (including both mir21-1 and miR21-2), miR-146a-5p, miR-146b-1-5p, and miR-193a-3p (including also mir193a-1-3p) were quantified through qPCR, after induction of ras expression and in the presence of drugs. For simplicity, the microRNAs under study are named miR-21, miR-146a, miR-146b, miR-193a from now on.

### The Increase of miR-21, miR-146a, and miR-193a Is Ras-Dependent

**FIGURE 2:**
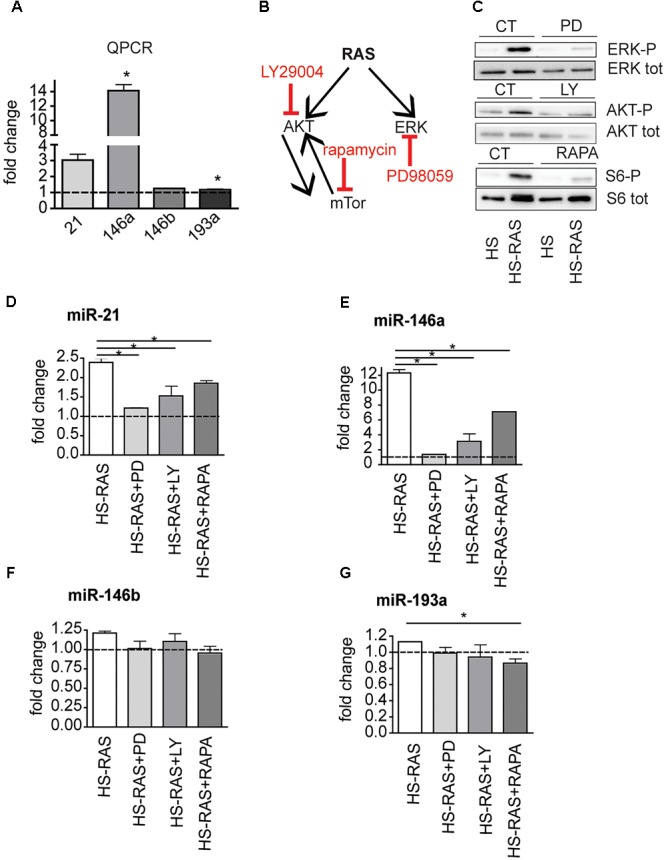
The increase of miR-21, miR-146a/b, and miR-193a is RAS-dependent. **(A)** Taqman QPCR analysis of miR-21, 146a, 146b, and 193a expression following ras upregulation (6 h after heat-shock induction), ^∗^*p* < 0.05. **(B)** Diagram showing the ras pathway inhibitors used and their targets. **(C)** Western Blot analysis of ERK-P, AKT-P, and S6-P ribosomal protein levels after the inhibitors treatment. **(D–G)** Taqman qPCR analysis in 3 dpf HS-RAS zebrafish treated with PD98059, LY29004 and rapamycin. The error bars represent the SEM of a triplicate experiment; two tailed Student’s test was used for analysis. ^∗^*p* < 0.05.

To investigate whether miR-21, miR-146a, miR-146b, and miR-193a are direct targets of ras, we analyzed their expression profiles using an inducible model, *Tg(hsp70l:EGFP-HRASV12)^io3^*, called HS-RAS, that expresses oncogenic ras upon heat shock. RNA was extracted from 3 dpf HS-RAS embryos, 6 h after a 30 min heat shock treatment at 37°C, when robust ras activation occurs (Santoriello et al., 2009). QPCR data show that miR-21 and miR-146a are significantly upregulated in Ras overexpressing larvae (**Figure 2A**), thus supporting the hypothesis that miR-21 and miR-146a represents early-response targets, likely to be directly induced by oncogenic Ras. MiR-193a and miR-146b did not show significant upregulation in response to ras; however, we performed the inhibitor experiments also on these two microRNAs as they had shown to be upregulated in the melanoma model (**Figure 1C**).

xs To identifying which signaling pathway(s) downstream of Ras induces overexpression of these microRNAs, we blocked specific pathways in HS-RAS larvae using small chemical inhibitors of ERK, AKT and mTor phosphorylation (**Figure 2B**). To check the efficacy of the inhibitors, we collected treated larvae at 3 dpf and performed western blot analysis for known targets of the three inhibitors. As shown in **Figure 2C** PD98059, rapamycin and LY29004 were able to decrease ERK-P, AKT-P and S6-P levels, respectively. Next we checked the levels of microRNAs in inhibitor treated larvae using qPCR. As shown in **Figures 2D,E**, induction of miR-21 and -146a expression was greatly attenuated by all three inhibitors and most robustly by the ERK inhibitor. MiR-146b levels were not affected by the drug treatments (**Figure 2F**) and miR-193a levels were reduced to statistically significant levels only when larvae were treated with rapamycin (**Figure 2G**). These data suggest that miR-21, miR-146a and to a less extent, miR-193a, are ras-responsive genes and their activation is regulated mostly by the MAPK/ERK and mTOR (for miR-193a) branches of ras signaling.

### Predicted Target of MicroRNAs 146a and 193a

As the function of miR-21 is widely studied in cancer (Frezzetti et al., 2011), and miR-146b was found not to respond to Ras signaling, in this study we focused on miR-146a and -193a, to clarify whether they have a role in melanomagenesis. A web-based target prediction algorithm [MicroCosmTargets, now incorporated in www.tools4mirs.org (Lukasik et al., 2016)], was used to identify potential targets of zebrafish miR-146a and miR-193a. The tool is based on the genome assembly ZV9^3^ and returned a few targets that were selected to contain seed sequences for miR- 146a, miR-193a or both, this last category included only *jmjd6*. We then used a web-based interaction-prediction algorithm for RNA molecules, IntaRNA^4^ (Mann et al., 2017) for the fast and accurate prediction of interactions between miR-146a and/or 193a with *jmjd6* mRNAs using the newest genome assembly, GRCz11^5^. This search confirmed the presence of seed sequences for miR-146a and -193a (see **Supplementary Figures S1A,B**).

### Jmjd6 Is a Target of miR-193a and miR-146a

**FIGURE 3:**
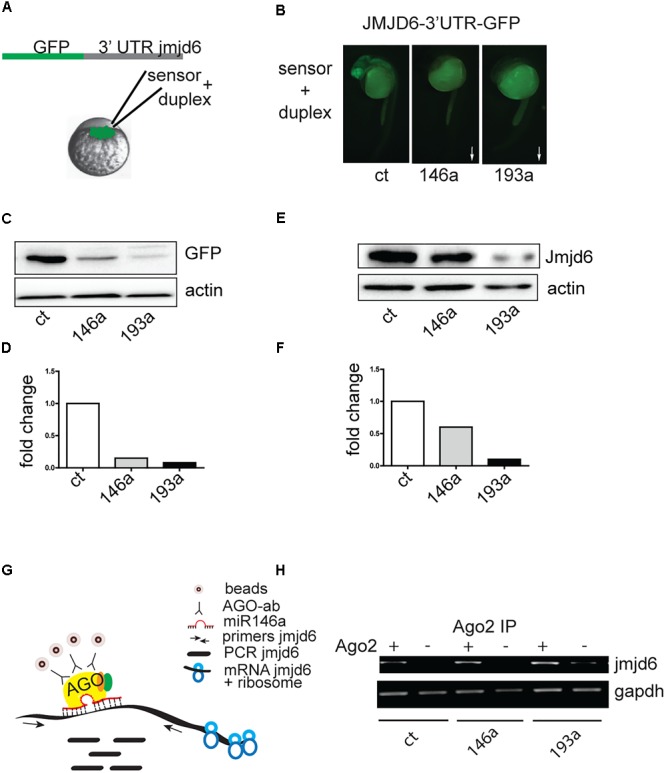
Jmjd6 is a target of miR-146a and miR-193a. **(A)** Effects of increasing microRNA levels on a Jmjd6-GFP sensor expression. Diagram of the construct used as sensor. **(B)** Representative images of 24 hpf zebrafish embryos injected with the Jmjd6-3′ UTR GFP sensor and the microRNA duplexes as indicated. Arrows illustrate the direction of the changes in expression. **(C)** Western blot analysis of GFP expression and **(D)** quantification of the changes in Jmjd6-3′ UTR GFP sensor levels upon microRNA overexpression. **(E)** Reduction of *Jmjd6* protein levels following miR-146a, and miR-193a duplex injection in 3 dpf embryos shown by Western Blot analysis. **(F)** Quantification of the Western Blot shown in **(E)**. **(G)** RIP (RNA immuno precipitation) diagram and **(H)** analysis of *jmjd6* transcripts in the RISC complex following duplex injections in 3 dpf embryos.

*Danio rerio jmjd6* has 1 transcript (*Ensemble, ENSDAR G00000102896*). The 3′ UTR region of *Jmjd6* contains miRNA recognition elements (MREs) for miR-146a (**Supplementary Figure S1A**), and MREs for miR-193a (**Supplementary Figure S1B**). To determine whether Jmjd6 is a bona fide target of miR-193a and miR-146a, we tested whether miR-193a and miR-146a expression affects *jmjd6* levels using an *in vivo* GFP reporter assay. The entire *jmjd6- 3′ UTR* was cloned downstream of green fluorescent protein (GFP) open reading frame (**Figure 3A**). *In vitro* synthesized mRNA from this construct was then injected into single-cell zebrafish embryos with or without miR-193a or mir-146a duplexes (**Figure 3A**), which mimic microRNA overexpression. The injection of the microRNA duplexes resulted in increased levels of microRNA (**Supplementary Figure S2A**) and caused mild or no phenotypes (**Supplementary Figure S2B**). The following day, GFP expression levels were monitored by fluorescence microscopy (**Figure 3B**) and by western blot analysis with an antibody against GFP (**Figures 3C,D**). In both assays, GFP levels were reduced in embryos injected with miR-193a or miR-146a duplexes. Duplex injection of either miR-146a or miR-193a also resulted in reduction of Jmjd6 protein levels (**Figures 3E,F**).

To validate the direct interaction between miR-146a and miR-193a with *jmjd6* mRNA, we performed RNA immunoprecipitation (RIP, **Figure 3G**). After immunoprecipitation (IP) with Ago2 antibody, which selectively enriched for RISC complex components (Ikeda et al., 2006), *jmjd6* transcripts were readily found in embryos injected with miR-146a and miR-193a duplexes (**Figure 3H**) suggesting that a physical interaction between *jmjd6* transcripts and specific microRNAs occurs in the RISC complex. These data confirmed the interaction between miR-146a and miR-193a with *jmjd6* mRNA.

As we found that *jmjd6* is a target of miR-146a and -193a, we investigated whether the level of jmjd6 was lower in Ras expressing larvae compared to controls. As shown in **Supplementary Figures S3A,B** heat-shock induced Ras overexpression results in down-regulation of *jmjd6* RNA level (**Supplementary Figure S3A**, column 2) and *jmjd6* protein levels (**Supplementary Figure S3B**, lane 2). To test if *jmjd6* down-regulation was due to increased expression of miR-146a and/or miR-193a, we reduced microRNA expression by injecting morpholinos (MO) specific to miR-146a or miR-193a and measured *jmjd6* mRNA and protein levels in morpholino injected HS-Ras embryos. All morpholinos were able to reduce expression levels of their targets (data not shown) and to increase the levels of *jmjd6* transcripts, repressed by ras, to levels similar to controls (**Supplementary Figure S3A**, columns 3–4) and protein levels (**Supplementary Figure S3B**, lanes 3–4), suggesting that ras-induced miRs are responsible for the downregulation of *jmjd6* levels observed in response to ras expression.

### Jmjd6 in Melanoma

**FIGURE 4:**
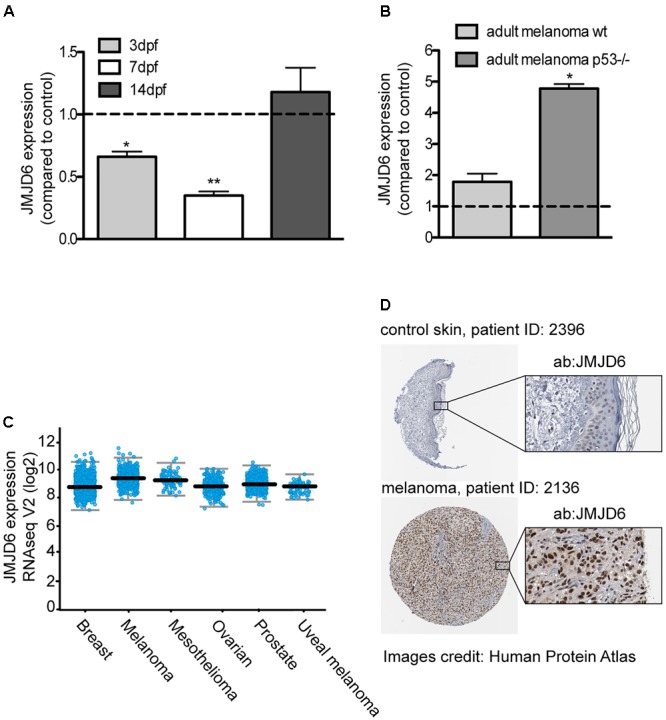
JMJD6 is up-regulated in zebrafish and human melanoma. **(A)** *Jmjd6* mRNA levels in 3, 7, 14 days kita-RAS zebrafish and **(B)** in adult melanoma developing in two different genetic backgrounds, as shown. The error bars represent the SEM of triplicate experiments; two tailed Student’s test was used for analysis. ^∗^*p* < 0.01. **(C)** Analysis of *JMJD6* expression in a variety of cancers from the cBioPortal database; every spot represents a case. **(D)** Expression of JMJD6 (brown nuclear staining) in control skin and in a malignant melanoma from the Protein Atlas website (https://www.proteinatlas.org/ENSG00000070495-JMJD6).

In our model of melanoma progression, where ras is overexpressed in melanocytes, *jmjd6* levels are reduced compared to control larvae at 3 and 7 dpf (**Figure 4A**). However, at 14 dpf, there is no significant difference in *jmjd6* levels between kita:Ras and controls. We then analyzed the levels of *jmjd6* expression in full-blown melanoma in two different genotypes: wild type (p53+/+) and p53-/-. In the latter background, melanomas developed earlier and were highly invasive from the earliest stages (Santoriello et al., 2010). QPCR analysis of *jmjd6* levels in six wild type and in three p53-/- cases showed significantly higher levels of expression of *jmjd6* in melanoma, especially in tumors developing in a p53-/- genetic background (**Figure 4B**).

Given the unexpected increase of *jmjd6* expression in melanoma, and its correlation with a more aggressive phenotype (Liu et al., 2017), to understand the clinical relevance of our findings, we queried online databases to investigate the expression levels of miR-146a, miR-193a and JMJD6 in melanoma samples from human patients. Data reported here on human melanoma are derived from publicly available resources, as stated in the following text and in the figure’s legend. While no changes in miR-193a expression were reported in melanoma, a couple of studies (GSE18509 and GSE31568, see dbDEMC at www.picb.ac.cn/dbDEMC/index.html) reported an increase of miR-146a in melanoma (not shown). Next, we analyzed the expression levels of JMJD6 in different types of cancers, including melanoma, using the website cBioPortal for Cancer Genomics^6^ (Cerami et al., 2012). As shown in **Figure 4C**, we found that JMJD6 is upregulated in different cancers, with melanoma being one of the cancer having higher levels of JMJD6 expression and high genomic alteration frequency (with amplification in almost 3% of cases, **Supplementary Figure S4A**). Looking carefully into the skin cutaneous melanoma TCGA dataset we found that the levels of *JMJD6* are upregulated in 16% of human cutaneous melanoma (72 out of 469 patients, **Supplementary Figure S4B**). Moreover, *JMJD6* expression levels are higher in the samples with HRAS and BRAF mutations compared to samples with *wild type* HRAS and BRAF (**Supplementary Figures S4C,D**), suggesting that *JMJD6* is upregulated by RAS signaling in these melanomas. Patients with alteration of *JMJD6* (mutations, amplifications, deep deletions, or multiple alterations) have a worse overall survival and disease/progression-free survival compared with patients with wild type JMJD6 (**Supplementary Figures S4E,F**), suggesting a key role of JMJD6 in disease progression.

Next, we investigated the levels of JMJD6 protein in human melanoma using the website Protein Atlas^7^ (Uhlen et al., 2017). The website reported medium (in three patients) or high (in nine patients) nuclear JMJD6 immunostaining in malignant melanoma. An example of a high JMJD6 immunostaining in melanoma is shown in comparison to control skin (where JMJD6 is not expressed) (**Figure 4D**) (Uhlen et al., 2017). These data suggest that the microRNA-mediated downregulation of *jmjd6* in the zebrafish progressive melanoma model is a transient event and at later stages of melanoma development or in more aggressive melanomas in fish and in human, *Jmjd6* is overexpressed.

### Expression of miR-Resistant Jmjd6 Promotes Ras-Induced Melanoma

**FIGURE 5:**
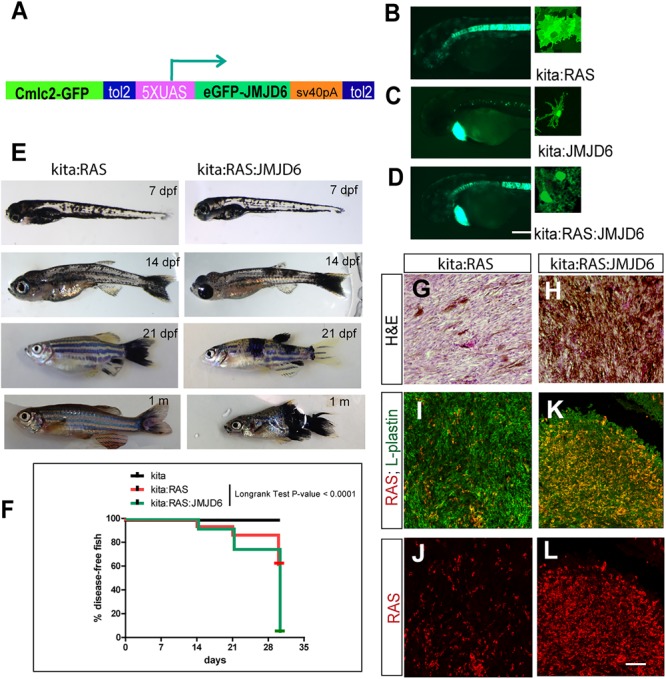
Expression of miR-resistant Jmjd6 promotes Ras-induced melanoma. **(A)** Schematic representation of the construct used to generate a transgenic line expressing microRNA-resistant Jmjd6 under the UAS promoter. Cmlc2-GFP is the cardiac myosin light chain promoter driving GFP expression in the heart as marker of transgenesis. **(B–D)** Examples of GFP staining in crosses between the UAS lines and the kita:Gal4 line (as indicated). Expression is visible in the notochord (Distel et al., 2009) and in melanocytes in all kita crosses **(B–D)**, and in the heart for the UAS:Jmjd6 line, **C,D**) GFP is localized to the nucleus for Jmjd6-GFP or the plasma membrane for eGFP-HRASV12. **(E)** Double or triple transgenic larvae and juveniles at the indicated stages of development. **(F)** Disease- free survival curve of the double or triple transgenic fish. *N* = 100 for kita; 321 for kita:Ras; 211 for kita:Ras:Jmjd6. Long-rank test: *p*-value < 0,0001. **(G,H)** H&E staining of representative melanoma sections from transgenic fish as indicated. **(I–L)** Immunostaining for L-plastin (red florescence) and GFP (only in **I–K**, indicating HRASV12, green fluorescence) in representative melanoma sections from transgenic fish as indicated. Calibration bar: 100 μm for **(B,D)**, 50 μm for **(I–L)**.

Given the dynamic changes in *Jmjd6* expression in the Ras-induced melanoma model in zebrafish, and the overall increase in expression in human melanoma, we wanted to clarify the role of Jmjd6 in melanoma with a gain of function approach. Therefore, we produced a transgenic line that expresses miR (146a and 193a) -resistant *jmjd6* in melanocytes. This was achieved by replacing the 3′ UTR of *jmjd6* in the transgenic construct with an artificial sequence (SV40 polyA, **Figure 5A**). With this construct, we generated a *tg(UAS:eGFP-jmjd6)* transgenic line using standard Tol2 transposase methods (Kawakami, 2004), and crossed it to kita:Gal4 fish. The double transgenic fish, designated as *kita:Jmjd6*, have no phenotype; however *eGFP-Jmjd6* expression was confirmed by kita driven GFP nuclear localization in melanocytes and notochord cells (**Figures 5B–D**). To study the effects of combined expression of oncogenic ras and Jmjd6 in melanoma we generated triple transgenic fish, *Et(kita:Gal4TA, UAS:mCherry)^hmz1^;Tg(UAS:eGFP-HRASV12)^io006^*; *Tg(UAS:eGFP-JMJD6)^ka202^* (designated as *kita/Ras/Jmjd6*, **Figure 5E**) and observe the fish for the development of melanoma at regular intervals, from 7 days to 1 month. As shown in the disease-free survival curve, 60% of double transgenic kita-ras fish show melanoma at 1 month (**Figure 5F**). However, triple transgenic fish *kita/Ras/Jmjd6* developed melanoma at an earlier stage (**Figures 5E,F**) and by 1 month of age, penetrance of melanoma was 95% (**Figure 5F**). The tumors appeared in multiple locations in the same fish (**Figure 5E**) and were more invasive, as judged by the body surface showing melanoma lesions and by histological analysis (**Figures 5G,H**).

We also noticed that Kita/Ras/Jmjd6 melanomas have massive infiltration of leukocytes (L-plastin+ cells), resembling macrophages and neutrophils for their shape, much higher than in to Kita/Ras melanomas (**Figures 5I–L**).

## Discussion

In this study we used a progressive model of melanoma in zebrafish to study the changes of microRNA expression at the onset of RAS-induced transformation and throughout progression of the disease. We found six microRNAs, which are upregulated as an early response to oncogenic RAS expression in three different models. As two of these microRNAs target Jmjd6 we investigated the significance of these interactions for melanoma progression. To our surprise we discovered that the target of these increased microRNAs, Jmjd6, is overexpressed in aggressive zebrafish melanoma. This suggested that overexpression of Jmjd6 promotes melanoma progression and that the increase of the microRNAs that downregulate Jmjd6 at the onset of Ras expression is part of a defensive response against the pro-oncogenic activity of Jmjd6. We validated this hypothesis by generating triple transgenic zebrafish that express micro-RNA resistant Jmjd6. Melanoma develop faster in the kita/ras/jmjd6 fish, supporting a pro-oncogenic role of Jmjd6. Further work is needed to clarify how Jmjd6 favors melanoma development.

Jmjd6 is a JumanjiC-domain containing protein, which has been endorsed for different functions. It was initially identified as a phosphatidylserine receptor (PSR) on the surface of phagocytes (Fadok et al., 2000). Ablation of Jmjd6 in mice, and morpholino downregulation of Jmjd6/PSR in zebrafish (Hong et al., 2004) causes abnormal development and leads to neonatal lethality in mouse (Böse et al., 2004; Kunisaki et al., 2004). However, many lines of evidence argue against a function of Jmjd6 as a PSR, primarily its nuclear localization (Cui et al., 2004; Tibrewal et al., 2007), and a number of newly reported nuclear activities. Subsequently, Jmjd6 was shown to function as an arginine demethylase, which removes repressive symmetric methylation marks from histone 2 (H2R3me2s) and histone 4 (H4R3me2s) arginines (Chang et al., 2007), but other groups have been unable to confirm the arginine demethylation activity of JMJD6 (Hahn et al., 2010). Webby et al. (2009) reported that JMJD6 acts as a lysyl-5-hydroxylase of the splice factor subunit U2A65. Silencing of JMJD6 expression in endothelial cells resulted in abnormal splicing of the VEGF receptor 1 (Boeckel et al., 2011). Meanwhile, a structural study suggested that methyl groups on single-stranded RNAs (ssRNAs) might be substrates of JMJD6 (Hong et al., 2010). More recently, a proteomic approach identified JMJD6 as one of a few binding partners of the bromodomain and extraterminal (BET) domain protein Brd4 (Rahman et al., 2011), which regulates gene expression through interaction with the cdk9 subunit of the positive transcription and elongation factor, pTEFb complex (Jang et al., 2005). Further studies lead to the identification of JMJD6 as the partner of BRD4 in binding distal enhancers known as anti-pause enhancers, which regulate release from transcriptional pausing in a large subset of transcriptional units which depend on long-range interactions (Liu et al., 2013). Here, the demethylase activity of JMJD6 on the snRNA, 7SK, results in the disassembly of the transcriptional repressive complex HEXIM1-7SK, releasing pTEFb from pausing (Liu et al., 2013). It would be interesting to study whether anti-pause enhancers, which are the substrates of the JMJD6-Brd4 interaction, are associated with pro-oncogenic gene expression in melanoma. Given the robust correlation between Jmjd6 overexpression and aggressive melanoma in different models and in human samples, it would be important to clarify if the pro-oncogenic function of JMJD6 depends on this transcriptional activity. More recently, Liu et al. (2017) have shown that the JMJD6 promotes melanomagenesis through the regulation of the alternative splicing of PAK1, a key MAPK signaling component. In recent years, also RNA binding abilities of Jmjd6 are emerging, perhaps its many functions, linked to arginine methylation of RNA binding proteins in transcription initiation complexes, spliceosome and ribosomes may be mediated by its RNA-binding abilities. Indeed Heim et al. (2014), showed that treatment with RNAse disrupt Jmjd6 nuclear localization.

In our study we found that two microRNAs induced by Ras (miR-146a and miR-193a) behave as tumor suppressors. MiR-146a and b are well-studied for their roles in immune responses. Induced by inflammation and infection through NF-kB signaling (Bhaumik et al., 2008), miR-146a, and to a lesser extent miR-146b, function in a negative feedback loop to downregulate several pro-inflammatory effectors. Indeed miR-146a has been implicated in attenuating pro-inflammatory responses (Saba et al., 2014), which could contribute in cancer to the elimination of early transformed cells by the immune system, and in reducing the metastatic potential of breast cancer cells (Bhaumik et al., 2008). Increase in expression of miR-146a was reported in unclassified melanoma samples (Philippidou et al., 2010), and found to suppress brain metastasis from melanoma in experimental models (Hwang et al., 2013). These findings support a tumor suppressor role for miR-146a in melanoma. Our study provides evidence that miR-146a is induced by the same MAPK/AKT pathway that sustains melanoma growth at the earliest stages of transformation, when it could exert an anti-melanoma function by repressing Jmjd6 and perhaps also pro-tumoral inflammation. The tumor suppressive activity of miR-193a has been related to its ability to inhibit cell proliferation and to promote apoptosis of cancer cells (Nakano et al., 2013; Mamoori et al., 2018). MiR-193a undergoes epigenetic silencing in acute myeloid leukemia by the AML1/ETO fusion protein (Li et al., 2013), where miR-193 was found to repress several pro-leukemogenic factors, including KIT. Moreover miR193a has been found to participate in a regulatory loop that controls p53 family member levels (Ory et al., 2011) thus further reinforcing the hypothesis that miR193a is a tumor suppressor. The finding that both microRNAs target Jmjd6 and that this regulation is conserved in zebrafish and human melanoma suggests that lowering JMJD6 levels in cancer is another function of the tumor suppressors miR-146a and miR-193a.

The mechanisms through which Jmjd6 promote melanoma progression is still unknown. Different approaches, including transcriptome, epigenome and spliceosome analysis, can help to gain insights into the pro-oncogenic activity of JMJD6. In these studies, the transgenic lines kita:Jmjd6 and kita/Ras/Jmjd6 will provide a source of GFP tagged Jmjd6 proteins expressed specifically in melanocytes. We were puzzled by the massive presence of L-plastin positive cells (leukocytes, possibly including neutrophils and macrophages) within the *kita/Ras/Jmjd6* melanomas, compared to *kita/Ras* melanomas. Neutrophils and eosinophil infiltrations in melanoma predict unfavorable disease outcome (Ding et al., 2018) whereas accumulation of dendritic cells and T-lymphocytes is positively correlated with survival. Therefore, the increase of L-plastin positive neutrophils and macrophages is an index of the aggressiveness of these tumors. It is intriguing that one of the microRNAs targeting Jmjd6 in this progressive model of melanoma is miR-146a, a well-known regulator of inflammatory responses. It would be interesting to investigate whether miR-146a levels differ in melanoma developing in *kita/Ras* (where they are increased according to RNA-Seq data) versus those developing in *kita/Ras/Jmjd6*.

In summary, this study has shown that several microRNAs are induced by RAS signaling in melanoma initiating cells at the onset of transformation. Induced miR-146a and -193a target Jmjd6, which is temporarily downregulated. At later stages of melanoma development, and in human malignant melanoma with unfavorable prognosis, Jmjd6 is overexpressed, and in a zebrafish model where melanocytes express both Ras and Jmjd6, melanomas are more aggressive. Therefore, Jmjd6 has pro-oncogenic activities and the initial downregulation mediated by microRNAs may be part of an anti-oncogenic response, which is induced by the same RAS oncogene.

## Materials and Methods

### Zebrafish Lines

Zebrafish were maintained and staged as described (Kimmel et al., 1995).

In addition to wild type AB fish, we used the following lines:

– *Et(kita:Gal4TA, UAS:mCherry)^hmz1^; Tg(UAS:eGFP-HRASV 12)^io006^* a double transgenic line in which oncogenic ras expression is regulated by the kita promoter and that develop melanoma (Santoriello et al., 2010), also crossed to *ZDF1 (p53^m214k/+^)*(Berghmans et al., 2005).
– *Tg(hsp70l:EGFP-HRAS_G12V)^io3^* an heat-inducible oncogene expressing line (Santoriello et al., 2009).
– *Et(zic4:Gal4TA4, UAS:mCherry)^hzm5;^Tg(UAS:eGFP-HRASV12)^io006^* a double transgenic line in which oncogenic ras expression is regulated by the zic4 promoter and that develop glioma (Mayrhofer et al., 2017).
– *Et(kita:Gal4TA, UAS:mCherry)^hmz1^; Tg(UAS:eGFP-Jmjd6)^ka202^;* and *Et(kita:Gal4TA, UAS:mCherry)^hmz1^; Tg(UAS:eGFP-HRASV12)^io006^; Tg(UAS:eGFP-Jmjd6)^ka202^*xs double and triple transgenic lines in which the expression of a microRNA resistant Jmjd6 is regulated by Gal4 expressed under the kita promoter alone (double) or together with HRASV12 (triple), generated in the course of this study. This study was carried out in accordance with the recommendations of the OPBA of the University of Trento on Animal Welfare. Experimental procedures on zebrafish were performed in accordance with the European law on Animal Protection and Authorization No. 75/2017-PR from the Italian Ministry of Health.

### miRNA Array

Custom-designed 8 × 15k microarray slides were ordered from Agilent Technologies. The 15k custom design was obtained from Edwin Cuppen and Eugene Berezikov (Hubrecht Institute, Utrecht, Netherlands) and has been submitted into the Gene Expression Omnibus (GEO) database (GPL 15403). The 15k design contained a duplicate of 7604 probes of 60-oligonucleotide length. The probes consisted of 2 × 22 nucleotide sequences antisense to mature miRNAs separated by a spacer of 8 nucleotides (CGATCTTT) and with a second spacer with the same sequence at the end. From 7604 probes 546 were designed for left (5′) and right (3′) arms of the hairpins of zebrafish miRNAs that are known in miRBase, while the remainder 7058 probes corresponded to predicted hairpin structures in the zebrafish genome that might include additional miRNAs but were not considered in this study. Total RNA, including microRNA, were extracted from 3-7-14 dpf wt or UAS-RAS larvae (driven by kita:Gal4, zic:Gal4, or hsp:Gal4), and from adult melanoma or wt fin tissue using miRNeasy Mini Kit^®^(Qiagen). Three biological replicates were obtained for each condition. For dual color hybridization of the Agilent chips miRNA samples from RAS transgenics were labeled with Hy3 and samples from control fish were labeled with Hy5 with miRCURY^TM^ LNA microRNA, Hy3^TM^/Hy5^TM^ Power Labeling kit (Exiqon) using 1 microgram of total RNA according to the manufacturer’s instructions. The dual color hybridization of the microarray chips was performed according to Agilent protocol GE2_105_Jan09 ^8^ for two-color microarray-based gene expression analysis except that hybridization and washing was performed at 37°C. The arrays were scanned with DNA Microarray Scanner G2565CA from Agilent Technologies. The arrays were scanned twice with 10% PMT and 100% PMT laser power. Microarray data was processed from raw data image files with Feature Extraction Software 9.5.3.1 (Agilent Technologies). The XDR function was used to extend the dynamic range. Processed data were subsequently imported into Rosetta Resolver 7.1 (Rosetta Biosoftware, Seattle, WA, United States) and subjected to default ratio error modeling. The raw data have been submitted to GEO under accession number GSE_37015. Values above 1.2 log2 Fold Changes and *p*-values < 0.01 in all three models were used as selection criteria.

### QPCR for MicroRNAs

To confirm microarray data, total RNA, including miRNA, were purified from 3 dpf embryos using miRNeasy Mini Kit^®^(Qiagen). Mature miRNA were reverse transcribed using specific primers mix for each miR to produce different cDNA for TaqMan^®^MicroRNA assay (30 ng of total mRNA for each reaction) (Applied Biosystems). Taqman probes were designed for the active strand (-5p) of miR-21, miR-146a, and miR-146b and for the active (-3p) strand of miR-193a. Real-time PCR reactions based on TaqMan reagent chemistry were performed in duplicate on ABI PRISM^®^7900HT Fast Real-Time PCR System (Applied Biosystems). The level of miRNA expression was measured using C_T_ (cycle threshold). For normalization, miR-133, which was unaffected by RAS overexpression, was taken as reference. Fold change was generated using the equation 2^-CT^. The list of oligos used and their sequences is provided in **Supplementary Table S2**.

### Inhibitor Treatment

Three dpf HS-RAS and AB embryos were incubated in 2ml E3 medium in a 12 well plate in the presence of the following inhibitors: 1 μg/ml PD98059 (Calbiochem), 15 μM LY294002 (Cell Signaling) and 1 μM Rapamycin (Tocris). After 2 h, embryos were heat-shocked at 39°C for 30 min. After 6 h 50 RAS-GFP+ or GFP- embryos were collected in Trizol or sample buffer (2% SDS, 10% glycerol, 60 mM Tris pH 6.8) for miRNA or protein extraction respectively.

### Western Blot and Antisera

Ten 3 dpf embryos were homogenized in 200 μl sample buffer (2% SDS, 10% glycerol, 60 mM Tris pH 6.8). 30 μg of total extract were resolved by SDS-PAGE, transferred to nitrocellulose and tested with the following antibodies: phospho-p44/42 (1:1000, Cell Signaling), p44/42 (1:1000, Cell Signaling), Phospho-AKT (1:1000, Cell Signaling), AKT (1:1000, Cell Signaling), Phospho-S6 (1:1000, Cell Signaling), S6 (Cell Signaling 1:1000), jmjd6 (Abcam, 1:1000), GFP (1:1000, Torrey Pines, United States), actin (1:5000, MP Biomedical).

### Manual Inspection of 3′ UTR of Candidate MicroRNA Targets

An initial analysis performed with MicroCosm (now incorporated in tools4mirs at https://tools4mirs.org/) which used a previous version of the zebrafish genome identified a few genes as potential targets of both miR146a and 193a. Manual inspection of the 3′ UTR regions of these genes using the GRCz11 release at Ensembl^5^ confirmed that only *jmjd6* 3′ UTR region contained sequences that match the seed regions of both microRNAs. We then used a web-based interaction-prediction algorithm for RNA molecules, IntaRNA^4^ (Mann et al., 2017) for the fast and accurate prediction of the interactions between miR-146a and/or 193a with *jmjd6* mRNAs (*Ensemble, ENSDARG00000102896*^5^). We followed the web-site instructions and used *jmjd6* and miR-146a-5p and -193a-3p sequences as input. The following parameters were used: minimum number of base pairs in seed: 7; temperature for energy computation: 37°C; energy parameter set (Vienna package), Turner Model 2004 (Lorenz et al., 2011); energy interaction levels < -8.

### Morpholino, Duplexes, and Plasmids

Morpholinos, including a standard control morpholino, were obtained from Gene Tools (United States), titrated to non-toxic concentrations and injected in a final volume of 2 nl per 1-cell embryo. A list of all morpholinos, their sequences and the concentration used is provided in **Supplementary Table S3**. Synthetic miR- duplexes controls and 146a were designed and ordered from SIGMA (United States). Synthetic miR- duplexes for miR-193a were purchased from Ambion. Duplexes were dissolved in RNAse free water and diluted using annealing buffer (30 mM HEPES-KOH pH 7.4, 10 mM KCl, 2 mM MgCl_2_, 50 mM NH_4_Ac) to a final concentration of 10 μM for miR-146a 5 μM for miR-193a. The solution was incubated for 1 min at 90°C, cooled down slowly to room temperature and injected in a final volume of 2 nl per 1-cell embryo. A list of all duplexes, their sequences and the concentration used is provided in **Supplementary Table S4**.

For Jmjd6 reporter construct, the whole *Jmjd6* (*Ensemble, ENSDARG00000102896*) 3′ UTRs was PCR amplified from cDNA using specific primers (**Supplementary Table S2**). The PCR product was subcloned into the pCS2:eGFP vector downstream of the GFP open reading frame and confirmed by sequencing.

### Generation of a Jmjd6 Transgenic Line

To express a microRNA resistant Jmjd6 in melanocytes, the full coding region excluding the stop codon of *Jmjd6* (*Ensemble, ENSDARG00000102896*) was PCR amplified and cloned into *pEntry5-no stop* (Invitrogen) and then recombined using gateway technology with a *pEnt5-4nrUAS* (generated by cloning four non-repetitive UAS elements into pENT5′ from Invitrogen), a *p3E-EGFPpA* (Tol2Kit clone n.366) + *pDestTol2CG2* (Tol2Kit clone n. 395) using gateway (Kwan and North, 2017). Clone numbers and sequences can be found at: http://chien.neuro.utah.edu/tol2kitwiki/index.php/Main_Page.

Final recombined clones were checked by sequencing. *4nrUAS:eGFP-Jmjd6* plasmid was injected in 1-cell embryos of the *Et(kita:Gal4TA, UAS:mCherry)^hmz1^* line to generate a transgenic line using standard Tol2 mediated transgenesis (Kawakami, 2004; Pase and Lieschke, 2009).

### Zebrafish Embryo Injections

Zebrafish embryos at the stage of 1–2 cells were injected with morpholinos against miRs or Jmjd6 diluted in double distilled, sterile H_2_O. The morpholino oligonucleotides were injected at a concentration of 5 ng/nl, in a volume of 2 nl/embryo. miR duplexes mimicking mature microRNA were injected following a described protocol (Pase and Lieschke, 2009), at a concentration of 10 μM for miR-146a and miR-146b and 5 μM for miR193a in a volume of 2 nl/embryo.

Sensor injections: mRNA encoding for eGFP carrying Jmjd6 3′ UTR was *in vitro* synthesized and injected in 1–2 cell zebrafish embryos at 100 pg/embryo, alone or in combination with duplexes or morpholinos.

### Imaging

Photographs of whole larvae were acquired with a Nikon S100 stereomicroscope equipped with epifluorescence and multiple filters. We used a Leica SP5 confocal for analysis of melanocytes expressing Ras-eGFP, Jmjd6-GFP, and L-plastin immunoreactivity.

### AGO2 – RIP

One hundred 3 dpf embryos injected or not with miR duplex were homogenized in ice-cold buffer A containing 50 mM Tris-HCl pH 7.5, 5 mM EDTA, 5 mM EGTA, complete proteinase inhibitor (Roche) and 1 U/μl RNAse inhibitor SUPERnase-IN (Ambion). Unbroken cells were removed at 100 g for 5 min at 4°C. NP40 was added at 0.5% (wt/vol) and samples incubated at 4°C for 15 min with rotation and centrifuged at 10000 × *g* for 15 min. SN was the transferred to a new tube and the protein were measured by BCA. 2 mg of protein were diluted with buffer A and incubated overnight at 4°C with 50 μl Dynabeads (Invitrogen) bound with 2 μg Ago2 antibody (Abcam) or IgG (negative control). The next morning beads were washed four times with 1 ml of washing buffer containing 50 mM Tris-HCl pH 7.4, 150 mM NaCl, 1 mM MgCl_2_ and 0.05% NP40. Beads were then resuspended in 1ml of washing buffer and 200 μl of beads were pelleted and resuspended in 50 ul sample buffer to test Ago2 by western blot (Abcam 1:1000). The remaining 800 ul were resuspend with 500 μl of trizol and RNA was extracted by RNeasy micro Kit (Qiagen). All the RNA was retro-transcribed using Vilo (Invitrogen) and 1 μl of cDNA was used for PCR using specific primers for *jmjd6* or the housekeeping gene, *gapdh*.

### Analysis of Data From cBioportal and Protein Atlas

Analysis of JMJD6 expression, correlation with HRAS or BRAF mutations, survival and disease/progression-free survival in patients was done using the dataset Skin Cutaneus Melanoma TCGA dataset in cBioportal^6^. The survival and disease/progression-free survival were done taking into account the following JMJD6 alterations: mutations, amplifications, deep deletions or multiple alterations.

We report here examples of JMJD6 protein localization in control skin (ID: 2396^9^) and in a malignant melanoma (ID: 2136^10^) from the Protein Atlas website.

## Author Contributions

VA and MM design the study, conducted experiments, and wrote the manuscript. VA, AO and AM performed the microarray analysis. SK, LS, and VG performed bioinformatics analysis. MS and AM provided conceptual insights.

## Conflict of Interest Statement

The authors declare that the research was conducted in the absence of any commercial or financial relationships that could be construed as a potential conflict of interest.

## Footnotes

1 http://www.sanger.ac.uk/genetics/CGP/cosmic/

2 http://cancergenome.nih.gov/

3 http://mar2015.archive.ensembl.org/Danio_rerio/Info/Index

4 http://rna.informatik.uni-freiburg.de/IntaRNA/Input.jsp

5 https://www.ensembl.org/Danio_rerio/Info/Index

6 http://www.cbioportal.org/

7 https://www.proteinatlas.org/

8 www.Agilent.com

9 https://www.proteinatlas.org/ENSG00000070495-JMJD6/tissue/skin

10 https://www.proteinatlas.org/ENSG00000070495-JMJD6/pathology/tissue/melanoma#img

## Supplementary Material

FIGURE S1 Output of IntaRNA website, showing interaction of microRNAs 146a **(A)** and 193a **(B)** with the 3′ UTR of jmjd6. An energy of -8 Kcal/mol or less is indicative of a stable interaction.

FIGURE S2 Injections of duplexes microRNAs cause increase of the corresponding microRNAs and mild developmental defects. **(A)** QPCR analysis of the expression levels of the microRNAs indicated. **(B)** All the other injected embryos developed without visible abnormalities.

FIGURE S3 Jmjd6 is down-regulated upon Ras expression. Jmjd6 mRNA **(A)** and protein levels **(B)** are downregulated by Ras overexpression (2nd lane) and rescued to normal levels by downregulating the increased microRNAs (lanes 3–4).

FIGURE S4 cBioPortal data of JMJD6. **(A)** Alteration frequency of JMJD6 in different type of cancers. **(B)** JMJD6 expression in melanoma patients; each bar represents a patient. Red bars: patients with upregulated JMJD6. Blue bars: patients with downregulated JMJD6. JMJD6 expression in HRAS **(C)** and BRAF **(D)** mutated melanoma. Overall survival **(E)** and disease/progression-free survival **(F)** in patients with (red curve) or without (blue curve) JMJD6 alterations.

TABLE S1 miRNA array results table. Column A: probe ID; column B: sequence of the probe; column C: Gene ID; column D: Gene symbol; Columns E–P: fold change and relative *p*-value of the samples as indicated. Fold change and *p*-value are calculated by comparing the miRNAs expression in kita-Ras larvae at 3, 7, and 14 dpf, HS-Ras, and zic at 3 dpf and adult melanoma with the respective control (AB for all the larvae and skin from adult AB wild type fish for the adult melanoma). MicroRNAs selected for further studies (see text for criteria used) and their expression values (log2FoldChanges and pValues) are highlighted.

TABLE S2 Oligo sequences. Sequences of the oligos used for qPCR, RIP assay, *jmjd6* 3′-UTR cloning and Gateway cloning of Jmjd6.

TABLE S3 Morpholino sequences. Sequences of the morpholinos used in the study.

TABLE S4 microRNA duplexes. Sequences of the miR control (miR-C) and miR-146a_l injected in the embryos. The sequence of miR-193a was not provided by the supplier (Ambion).

## References

[B1] Berghmans S. Murphey R. D. Wienholds E. Neuberg D. Kutok J. L. Fletcher C. D. M. (2005). tp53 mutant zebrafish develop malignant peripheral nerve sheath tumors. *Proc. Natl. Acad. Sci. U.S.A.* 102 407–412. 10.1073/pnas.0406252102 15630097 PMC544293

[B2] Bhaumik D. Scott G. K. Schokrpur S. Patil C. K. Campisi J. Benz C. C. (2008). Expression of microRNA-146 suppresses NF-kappaB activity with reduction of metastatic potential in breast cancer cells. *Oncogene* 27 5643–5647. 10.1038/onc.2008.171 18504431 PMC2811234

[B3] Boeckel J.-N. Guarani V. Koyanagi M. Roexe T. Lengeling A. Schermuly R. T. (2011). Jumonji domain-containing protein 6 (Jmjd6) is required for angiogenic sprouting and regulates splicing of VEGF-receptor 1. *Proc. Natl. Acad. Sci. U.S.A.* 108 3276–3281. 10.1073/pnas.1008098108 21300889 PMC3044381

[B4] Böse J. Gruber A. D. Helming L. Schiebe S. Wegener I. Hafner M. (2004). The phosphatidylserine receptor has essential functions during embryogenesis but not in apoptotic cell removal. *J. Biol.* 3:15. 10.1186/jbiol10 15345036 PMC549712

[B5] Cerami E. Gao J. Dogrusoz U. Gross B. E. Sumer S. O. Aksoy B. A. (2012). The cBio cancer genomics portal: an open platform for exploring multidimensional cancer genomics data. *Cancer Discov.* 2 401–404. 10.1158/2159-8290.CD-12-0095 22588877 PMC3956037

[B6] Chang B. Chen Y. Zhao Y. Bruick R. K. (2007). JMJD6 is a histone arginine demethylase. *Science* 318 444–447. 10.1126/science.1145801 17947579

[B7] Chin L. Artandi S. E. Shen Q. Tam A. Lee S. L. Gottlieb G. J. (1999a). deficiency rescues the adverse effects of telomere loss and cooperates with telomere dysfunction to accelerate carcinogenesis. *Cell* 97 527–538. 10338216 10.1016/s0092-8674(00)80762-x

[B8] Chin L. Tam A. Pomerantz J. Wong M. Holash J. Bardeesy N. (1999b). Essential role for oncogenic Ras in tumour maintenance. *Nature* 400 468–472. 10.1038/22788 10440378

[B9] Cui P. Qin B. Liu N. Pan G. Pei D. (2004). Nuclear localization of the phosphatidylserine receptor protein via multiple nuclear localization signals. *Exp. Cell Res.* 293 154–163. 10.1016/j.yexcr.2003.09.023 14729065

[B10] Ding Y. Zhang S. Qiao J. (2018). Prognostic value of neutrophil-to-lymphocyte ratio in melanoma: evidence from a PRISMA-compliant meta-analysis. *Medicine* 97:e11446. 10.1097/MD.0000000000011446 30045267 PMC6078713

[B11] Distel M. Wullimann M. F. Köster R. W. (2009). Optimized Gal4 genetics for permanent gene expression mapping in zebrafish. *Proc. Natl. Acad. Sci. U.S.A.* 106 13365–13370. 10.1073/pnas.0903060106 19628697 PMC2726396

[B12] Fadok V. A. Bratton D. L. Rose D. M. Pearson A. Ezekewitz R. A. Henson P. M. (2000). A receptor for phosphatidylserine-specific clearance of apoptotic cells. *Nature* 405 85–90. 10.1038/35011084 10811223

[B13] Frezzetti D. De Menna M. Zoppoli P. Guerra C. Ferraro A. Bello A. M. (2011). Upregulation of miR-21 by Ras *in vivo* and its role in tumor growth. *Oncogene* 30 275–286. 10.1038/onc.2010.416 20956945

[B14] Hahn P. Wegener I. Burrells A. Böse J. Wolf A. Erck C. (2010). Analysis of Jmjd6 cellular localization and testing for its involvement in histone demethylation. *PLoS One* 5:e13769. 10.1371/journal.pone.0013769 21060799 PMC2966431

[B15] Harrandah A. M. Mora R. A. Chan E. K. L. (2018). Emerging microRNAs in cancer diagnosis, progression, and immune surveillance. *Cancer Lett.* 438 126–132. 10.1016/j.canlet.2018.09.019 30237038

[B16] Heim A. Grimm C. Müller U. Häußler S. Mackeen M. M. Merl J. (2014). Jumonji domain containing protein 6 (Jmjd6) modulates splicing and specifically interacts with arginine-serine-rich (RS) domains of SR- and SR-like proteins. *Nucleic Acids Res.* 42 7833–7850. 10.1093/nar/gku488 24914048 PMC4081092

[B17] Hong J.-R. Lin G.-H. Lin C. J.-F. Wang W.-P. Lee C.-C. Lin T.-L. (2004). Phosphatidylserine receptor is required for the engulfment of dead apoptotic cells and for normal embryonic development in zebrafish. *Development* 131 5417–5427. 10.1242/dev.01409 15469976

[B18] Hong X. Zang J. White J. Wang C. Pan C.-H. Zhao R. (2010). Interaction of JMJD6 with single-stranded RNA. *Proc. Natl. Acad. Sci. U.S.A.* 107 14568–14572. 10.1073/pnas.1008832107 20679243 PMC2930430

[B19] Hwang S. H. Lee B.-H. Kim H.-J. Cho H.-J. Shin H.-C. Im K.-S. (2013). Suppression of metastasis of intravenously-inoculated B16/F10 melanoma cells by the novel ginseng-derived ingredient, gintonin: involvement of autotaxin inhibition. *Int. J. Oncol.* 42 317–326. 10.3892/ijo.2012.1709 23174888

[B20] Ikeda K. Satoh M. Pauley K. M. Fritzler M. J. Reeves W. H. Chan E. K. L. (2006). Detection of the argonaute protein Ago2 and microRNAs in the RNA induced silencing complex (RISC) using a monoclonal antibody. *J. Immunol. Methods* 317 38–44. 10.1016/j.jim.2006.09.010 17054975 PMC1913063

[B21] Jang M. K. Mochizuki K. Zhou M. Jeong H.-S. Brady J. N. Ozato K. (2005). The bromodomain protein Brd4 is a positive regulatory component of P-TEFb and stimulates RNA polymerase II-dependent transcription. *Mol. Cell* 19 523–534. 10.1016/j.molcel.2005.06.027 16109376

[B22] Johnson L. Mercer K. Greenbaum D. Bronson R. T. Crowley D. Tuveson D. A. (2001). Somatic activation of the K-ras oncogene causes early onset lung cancer in mice. *Nature* 410 1111–1116. 10.1038/35074129 11323676

[B23] Kawakami K. (2004). Transgenesis and gene trap methods in zebrafish by using the Tol2 transposable element. *Methods Cell Biol.* 77 201–222. 10.1016/S0091-679X(04)77011-9 15602913

[B24] Kimmel C. B. Ballard W. W. Kimmel S. R. Ullmann B. Schilling T. F. (1995). Stages of embryonic development of the zebrafish. *Dev. Dyn.* 203 253–310. 10.1002/aja.1002030302 8589427

[B25] Kunisaki Y. Masuko S. Noda M. Inayoshi A. Sanui T. Harada M. (2004). Defective fetal liver erythropoiesis and T lymphopoiesis in mice lacking the phosphatidylserine receptor. *Blood* 103 3362–3364. 10.1182/blood-2003-09-3245 14715629

[B26] Kwan W. North T. E. (2017). Netting novel regulators of hematopoiesis and hematologic malignancies in zebrafish. *Curr. Top. Dev. Biol.* 124 125–160. 10.1016/bs.ctdb.2016.11.005 28335858

[B27] Li Y. Gao L. Luo X. Wang L. Gao X. Wang W. (2013). Epigenetic silencing of microRNA-193a contributes to leukemogenesis in t(8;21) acute myeloid leukemia by activating the PTEN/PI3K signal pathway. *Blood* 121 499–509. 10.1182/blood-2012-07-444729 23223432

[B28] Liu W. Ma Q. Wong K. Li W. Ohgi K. Zhang J. (2013). Brd4 and JMJD6-associated anti-pause enhancers in regulation of transcriptional pause release. *Cell* 155 1581–1595. 10.1016/j.cell.2013.10.056 24360279 PMC3886918

[B29] Liu X. Si W. Liu X. He L. Ren J. Yang Z. (2017). JMJD6 promotes melanoma carcinogenesis through regulation of the alternative splicing of PAK1, a key MAPK signaling component. *Mol. Cancer* 16:175. 10.1186/s12943-017-0744-2 29187213 PMC5708181

[B30] Lorenz R. Bernhart S. H. Höner Zu Siederdissen C. Tafer H. Flamm C. Stadler P. F. (2011). ViennaRNA Package 2.0. *Algorithms Mol. Biol.* 6:26. 10.1186/1748-7188-6-26 22115189 PMC3319429

[B31] Lukasik A. Wójcikowski M. Zielenkiewicz P. (2016). Tools4miRs – one place to gather all the tools for miRNA analysis. *Bioinformatics* 32 2722–2724. 10.1093/bioinformatics/btw189 27153626 PMC5013900

[B32] Malumbres M. Barbacid M. (2003). RAS oncogenes: the first 30 years. *Nat. Rev. Cancer* 3 459–465. 10.1038/nrc1097 12778136

[B33] Mamoori A. Gopalan V. Lam A. K.-Y. (2018). Role of miR-193a in cancer: complexity and factors control the pattern of its expression. *Curr. Cancer Drug Targets* 18 618–628. 10.2174/1568009618666180308105727 29521232

[B34] Mann M. Wright P. R. Backofen R. (2017). IntaRNA 2.0: enhanced and customizable prediction of RNA-RNA interactions. *Nucleic Acids Res.* 45 W435–W439. 10.1093/nar/gkx279 28472523 PMC5570192

[B35] Mayrhofer M. Gourain V. Reischl M. Affaticati P. Jenett A. Joly J.-S. (2017). A novel brain tumour model in zebrafish reveals the role of YAP activation in MAPK- and PI3K-induced malignant growth. *Dis. Model Mech.* 10 15–28. 10.1242/dmm.026500 27935819 PMC5278524

[B36] Nakano H. Yamada Y. Miyazawa T. Yoshida T. (2013). Gain-of-function microRNA screens identify miR-193a regulating proliferation and apoptosis in epithelial ovarian cancer cells. *Int. J. Oncol.* 42 1875–1882. 10.3892/ijo.2013.1896 23588298 PMC3699598

[B37] Ory B. Ramsey M. R. Wilson C. Vadysirisack D. D. Forster N. Rocco J. W. (2011). A microRNA-dependent program controls p53-independent survival and chemosensitivity in human and murine squamous cell carcinoma. *J. Clin. Invest.* 121 809–820. 10.1172/JCI43897 21293058 PMC3026726

[B38] Pase L. Lieschke G. J. (2009). Validating microRNA target transcripts using zebrafish assays. *Methods Mol. Biol.* 546 227–240. 10.1007/978-1-60327-977-2_14 19378107

[B39] Philippidou D. Schmitt M. Moser D. Margue C. Nazarov P. V. Muller A. (2010). Signatures of microRNAs and selected microRNA target genes in human melanoma. *Cancer Res.* 70 4163–4173. 10.1158/0008-5472.CAN-09-4512 20442294

[B40] Rahman S. Sowa M. E. Ottinger M. Smith J. A. Shi Y. Harper J. W. (2011). The Brd4 extraterminal domain confers transcription activation independent of pTEFb by recruiting multiple proteins, including NSD3. *Mol. Cell. Biol.* 31 2641–2652. 10.1128/MCB.0134110 21555454 PMC3133372

[B41] Saba R. Sorensen D. L. Booth S. A. (2014). MicroRNA-146a: a dominant, negative regulator of the innate immune response. *Front. Immunol.* 5:578. 10.3389/fimmu.2014.00578 25484882 PMC4240164

[B42] Santoriello C. Deflorian G. Pezzimenti F. Kawakami K. Lanfrancone L. d’Adda di Fagagna F. (2009). Expression of H-RASV12 in a zebrafish model of Costello syndrome causes cellular senescence in adult proliferating cells. *Dis. Model Mech.* 2 56–67. 10.1242/dmm.001016 19132118 PMC2615164

[B43] Santoriello C. Gennaro E. Anelli V. Distel M. Kelly A. Köster R. W. (2010). Kita driven expression of oncogenic HRAS leads to early onset and highly penetrant melanoma in zebrafish. *PLoS One* 5:e15170. 10.1371/journal.pone.0015170 21170325 PMC3000817

[B44] Tibrewal N. Liu T. Li H. Birge R. B. (2007). Characterization of the biochemical and biophysical properties of the phosphatidylserine receptor (PS-R) gene product. *Mol. Cell. Biochem.* 304 119–125. 10.1007/s11010-007-9492-8 17534701

[B45] Uhlen M. Zhang C. Lee S. Sjöstedt E. Fagerberg L. Bidkhori G. (2017). A pathology atlas of the human cancer transcriptome. *Science* 357:eaan2507. 10.1126/science.aan2507 28818916

[B46] Webby C. J. Wolf A. Gromak N. Dreger M. Kramer H. Kessler B. (2009). Jmjd6 catalyses lysyl-hydroxylation of U2AF65, a protein associated with RNA splicing. *Science* 325 90–93. 10.1126/science.1175865 19574390

