# Supplementary material for: Ras-Induced miR-146a and 193a Target Jmjd6 to Regulate Melanoma Progression
Source: Front Genet. 2018 Dec 18;9:675. doi: 10.3389/fgene.2018.00675 (PMC6305343; doi:10.3389/fgene.2018.00675)
Supplement: FIGURE S1 — Output of IntaRNA website, showing interaction of microRNAs 146a (A) and 193a (B) with the 3′ UTR of jmjd6. An energy of -8 Kcal/mol or less is indicative of a stable interaction. [file Data_Sheet_1.PDF]

A miR-146a-5p

```
3’UTR JMJD6 zebrafish
           184              199
             |              |
5'-CAA...GACG           U    CAGG...AAU-3'
             CUGUUUGUGGA UUCA
             |::|:|:|||| ||||
             GGUAGAUACCU AAGU
          3'-           U    CAAGAGU-5'
             |              |
            23              8
dre-miR-146a MIMAT0001843

interaction seq1   = 184 -- 199
interaction seq2   = 8 -- 23

interaction energy = -8.67217 kcal/mol
```

B miR-193a-3p

```
3’UTR JMJD6 zebrafish
          4367            4381
             |              |
5'-CAA...UACG       G       GUAA...AAU-3'
             UGGGGUU UGUGGGC
             ||||::| |||:|||
             ACCCUGA ACAUCCG
        3'-UG       A       GUCAA-5'
             |              |
            20              6
dre-miR-193a-3p MIMAT0001856

interaction seq1   = 4367 -- 4381
interaction seq2   = 6 -- 20

    interaction energy = -9.47894 kcal/mol
```

Figure S1 - Output of IntaRNA website, showing interaction of microRNAs 146a (A) and 193a (B) with the 3’UTR of jmjd6. An energy of -8 Kcal/mol or less is indicative of a stable interaction.

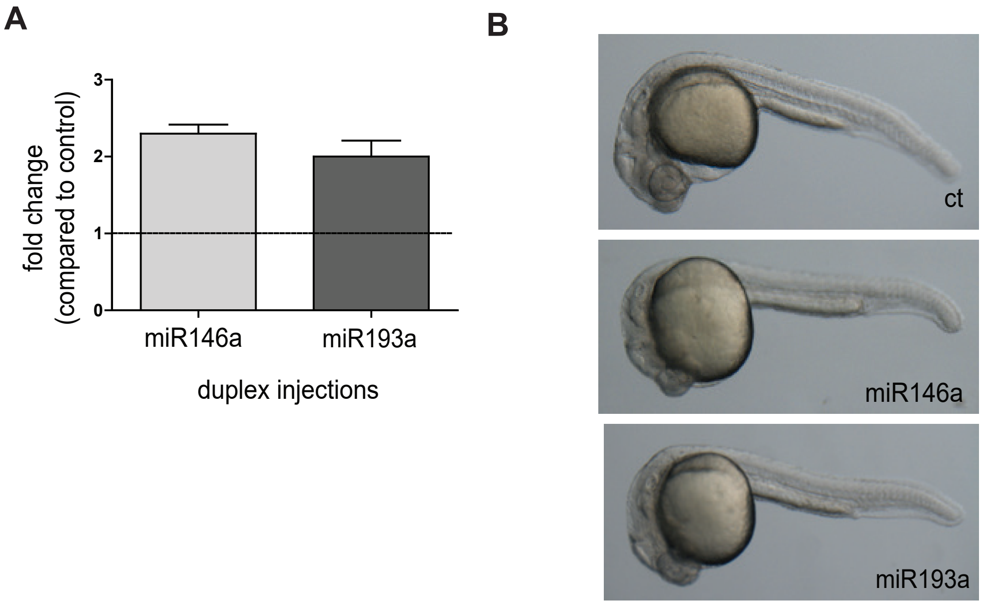

Figure S2 - Injections of duplexes microRNAs cause increase of the corresponding microRNAs and mild developmental defects.
(A) QPCR analysis of the expression levels of the microRNAs indicated.
(B) All the other injected embryos developed without visible abnormalities.

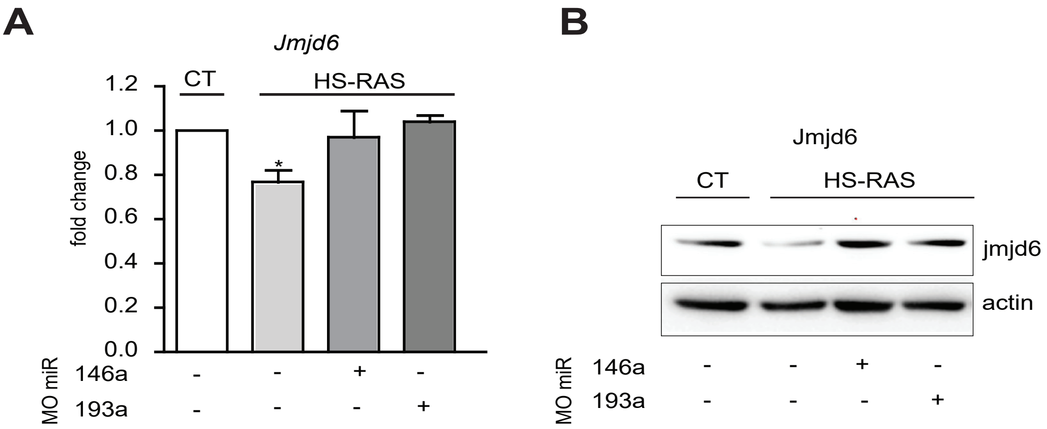

Figure S3. Jmjd6 is down-regulated upon Ras expression.
Jmjd6 mRNA (A) and protein levels (B) are downregulated by Ras overexpression (2nd lane) and rescued to normal levels by downregulating the increased microRNAs (lanes 3-4).

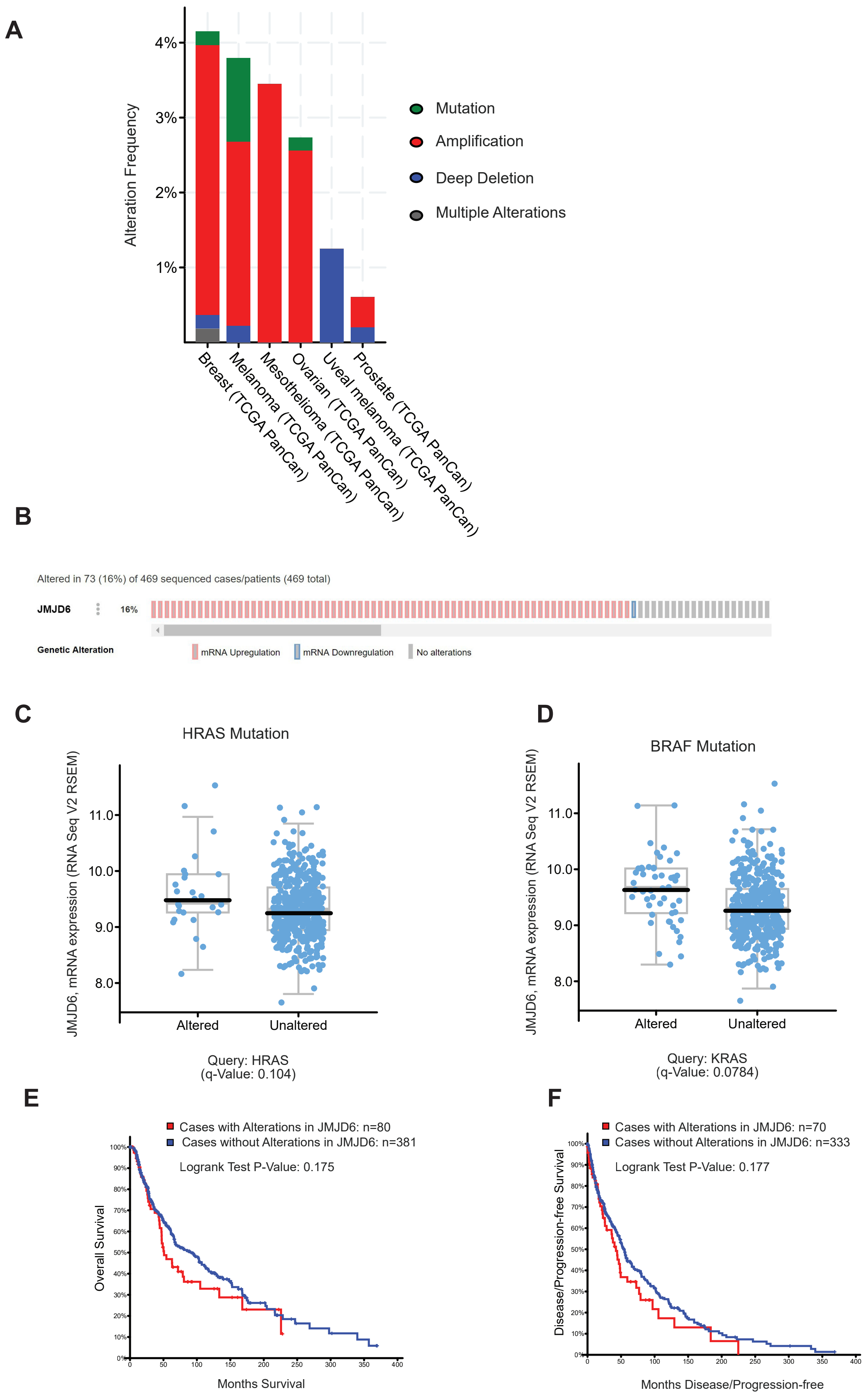

Figure S4. cBioPortal data of JMJD6. (A) Alteration frequency of JMJD6 in different type of cancers. (B) JMJD6 expression in melanoma patients; each bar represents a patient. Red bars: patients with upregulated JMJD6. Blue bars: patients with downregulated JMJD6. (C) and (D) JMJD6 expression in HRAS (C) and BRAF (D) mutated melanoma. Overall survival (E) and disease/progression-free survival in patients with (red curve) or without (blue curve) JMJD6 alterations.
