# Supplementary material for: Ras-Induced miR-146a and 193a Target Jmjd6 to Regulate Melanoma Progression
Source: Front Genet. 2018 Dec 18;9:675. doi: 10.3389/fgene.2018.00675 (PMC6305343; doi:10.3389/fgene.2018.00675)
Supplement: TABLE S2 — Oligo sequences. Sequences of the oligos used for qPCR, RIP assay, jmjd6 3′-UTR cloning and Gateway cloning of Jmjd6. [file Data_Sheet_2.docx]

**Supplementary Table 2: Oligo Sequences**

| **gene** | **Purpose** | **Oligo sequence** |
| --- | --- | --- |
| **zf_jmjd6_F** | QPCR/RIP | AGCTCCTCTGACAGCGACTC |
| **zf_jmjd6_R** | QPCR/RIP | CGCAAGCACACTTCTTGGTA |
| **zf_act_F** | QPCR | CGAGCAGGAGATGGGAACC |
| **zf_act_R** | QPCR | CAACGGAAACGCTCATTGC |
| **zf_GAPDH_F** | QPCR/RIP | GTGGAGTCTACTGGTGTCTTC |
| **zf_GAPDH_R** | QPCR/RIP | GTGCAGGAGGCATTGCTTACA |
| **zf_jmjd6_F** | 3’UTR cloning | GCCTCGAGCAAAACTGAGGAACCTGAACTA |
| **zf_jmjd6_F** | 3’UTR cloning | GCTCTAGACCCTCTATTTACTCCCTCAAGC |
| **zf_jmjd6_F** | ORF cloning | CACCATGAACCATAAAAGCAAGAA |
| **Zf_jmjd6_R** | ORF cloning | CCGGGATGAACTGCGCTCTT |

**Supplementary Table 3**: **Morpholino sequences**

| **Gene** | **Sequence (5’-3’)** | **concentration** |
| --- | --- | --- |
| dre-mir-146a | ACCATCTATGGAATTCAGTTCTCAG | 5ng/nl |
| dre-mir-193a | ACTGGGACTTTGTAGGCCAGTTGAG | 5ng/nl |

**Supplementary Table 4**: **microRNA duplexes**

| **name** | **Sequence (5’-3’)** | **concentration** |
| --- | --- | --- |
| miR-C | ugauaaucagaugaaguaCT | 10fmol/nl |
| miR-C* | uacuucaucugauuaugaTT | 10fmol/nl |
| miR-146a_l | 5’ugagaacugaauuccauagauGG 3’ | 10fmol/nl |
| miR-146a_l* | 5’aucuauggaauucaguucuaaTT 3’ | 10fmol/nl |
| miR-193a | has-miR-193 Pre-miR miRNA precursor (Ambion) | 5fmol/nl |
